# Supplementary material for: The Application of DTCWT on MRI-Derived Radiomics for Differentiation of Glioblastoma and Solitary Brain Metastases
Source: J Pers Med. 2022 Aug 3;12(8):1276. doi: 10.3390/jpm12081276 (PMC9409920; doi:10.3390/jpm12081276)
Supplement: Supplementary file 1 [file jpm-12-01276-s001.zip › jpm-1786576-supplementary.pdf]

Supplementary Materials

Table S1. The definition of the employed metrics.

$\text{Energy} = \frac{1}{N^2}\sum_{i=1}^{N}\sum_{j=1}^{N} {P_{i,j}}^2$
$\text{Mean } (\mu) = \frac{1}{N^2}\sum_{i=1}^{N}\sum_{j=1}^{N} P_{i,j}$
$\text{Standard Deviation } (S) = \sqrt{\frac{1}{N^2-1}\sum_{i=1}^{N}\sum_{j=1}^{N} (P_{i,j}-\mu)^2}$
$\text{Skewness} = \frac{1}{N^2}\sum_{i=1}^{N}\sum_{j=1}^{N} (\frac{P_{i,j}-\mu}{S})^3$
$\text{Kurtosis} = \frac{1}{N^2}\sum_{i=1}^{N}\sum_{j=1}^{N} (\frac{P_{i,j}-\mu}{S})^4$
$\text{Coefficient of Variation (CV)} = \frac{S}{\mu}$
$\text{Entropy} = -\sum_{i=1}^{N}\sum_{j=1}^{N} (P_{i,j} \times \log_2(P_{i,j}))$

Table S2. The relevant items from the Image Biomarker Standardization Initiative (IBSI) guideline.

Topic	Modality	Item	Description
			Patient
Region of interest		1	Describe the region of interest that is being imaged. *
Patient preparation		2a	Describe specific instructions given to patients prior to image acquisition, e.g., fasting prior to imaging. *
		2b	Describe administration of drugs to the patient prior to image acquisition, e.g., muscle relaxants. *
		2c	Describe the use of specific equipment for patient comfort during scanning, e.g., ear plugs. *
Contrast agent		4a	Describe which contrast agent was administered to the patient. *
		4b	Describe the administration method. *
		4c	Describe the injected quantity of contrast agent. *
		4d	Describe the uptake time prior to image acquisition. *
		4e	Describe how competing substance levels were controlled. **
Comorbidities		5	Describe if the patients have comorbidities that affect imaging. *
			Acquisition
Acquisition protocol		6	Describe whether a standard imaging protocol was used, and where its description may be found. *
Scanner type		7	Describe the scanner type(s) and vendor(s) used in the study. *
Imaging modality		8	Clearly state the imaging modality that was used in the study, e.g., CT, MRI. *
Static/dynamic scans		9a	State if the scans were static or dynamic. *
Scanner calibration		10	Describe how and when the scanner was calibrated. **
Patient instructions		11	Describe specific instructions given to the patient during acquisition, e.g., breath holding. **
Anatomical motion correction		12	Describe the method used to minimise the effect of anatomical motion. *

Scan duration		13	Describe the duration of the complete scan or the time per bed position. *
RF coil	MRI	17	Describe what kind RF coil used for acquisition, including vendor. *
Scanning sequence	MRI	18a	Describe which scanning sequence was acquired. *
	MRI	18b	Describe which sequence variant was acquired. *
	MRI	18c	Describe which scan options apply to the current sequence, e.g., flow compensation, cardiac gating. *
Repetition time	MRI	19	Describe the time in ms between subsequent pulse sequences. *
Echo time	MRI	20	Describe the echo time in ms. *
Echo train length	MRI	21	Describe the number of lines in k-space that are acquired per excitation pulse. **
Inversion time	MRI	22	Describe the time in ms between the middle of the inverting RF pulse to the middle of the excitation pulse. *
Flip angle	MRI	23	Describe the flip angle produced by the RF pulses. *
Acquisition type	MRI	24	Describe the acquisition type of the MRI scan, e.g., 3D. *
k-space traversal	MRI	25	Describe the acquisition trajectory of the k-space. **
Number of averages/ excitations	MRI	26	Describe the number of times each point in k-space is sampled. *
Magnetic field strength	MRI	27	Describe the nominal strength of the MR magnetic field. *
			Reconstruction
In-plane resolution		28	Describe the distance between pixels, or alternatively the field of view and matrix size. *
Image slice thickness		29	Describe the slice thickness. *
Image slice spacing		30	Describe the distance between image slices. *
Reconstruction method	MRI	35a	Describe the reconstruction method used to reconstruct the image from the k-space information. **
	MRI	35b	Describe any artifact suppression methods used during reconstruction to suppress artifacts due to undersampling of k-space. **
Diffusion-weighted imaging	DWI-MRI	36	Describe the b-values used for diffusion-weighting. *
			Image Registration
Registration method		37	Describe the method used to register multi-modality imaging. **
			Image Processing—Data Conversion
ADC computation	DWI-MRI	39	Describe how apparent diffusion coefficient (ADC) values were calculated. *
Other data conversions		40	Describe any other conversions that are performed to generate e.g., perfusion maps. *
			Image Processing—Post-acquisition Processing
Anti-aliasing		41	Describe the method used to deal with anti-aliasing when down-sampling during interpolation. **
Noise suppression		42	Describe methods used to suppress image noise. **
Skull stripping	MRI (brain)	44	Describe method used to perform skull stripping. **
Non-uniformity correction	MRI	45	Describe the method and settings used to perform non-uniformity correction. **
Intensity normalisation		46	Describe the method and settings used to normalise intensity distributions within a patient or patient cohort. **
Other post-acquisition processing methods		47	Describe any other methods that were used to process the image and are not mentioned separately in this list. *
			Segmentation
Segmentation method		48a	Describe how regions of interest were segmented, e.g., manually. *
		48b	Describe the number of experts, their expertise and consensus strategies for manual delineation. *
		48d	Describe which image was used to define segmentation in case of multi-modality imaging. *
Conversion to mask		49	Describe the method used to convert polygonal or mesh-based segmentations to a voxel-based mask. **
			Image Processing—ROI Interpolation
Interpolation method		53	Describe which interpolation algorithm was used to interpolate the region of interest mask. **
Partially masked voxels		54	Describe how partially masked voxels after interpolation are handled. **

Image Processing—Re-segmentation		
Re-segmentation methods	55	Describe which methods and settings are used to re-segment the ROI intensity mask. **
Image Processing—Discretisation		
Discretisation method	56a	Describe the method used to discretise image intensities. *
	56b	Describe the number of bins (FBN) or the bin size (FBS) used for discretisation. *
	56c	Describe the lowest intensity in the first bin for FBS discretisation. **
Radiomics Feature Computation		
Feature set	58	Describe which set of radiomics features is computed and refer to their definitions or provide these. *
IBSI compliance	59	State if the software used to extract the set of features is able to reproduce the IBSI feature reference values. **
Robustness	60	Describe how robustness of the features was assessed, e.g., test-retest analysis. **
Software availability	61	Describe which software and version were used to compute features. *
Radiomics Feature Computation—Texture Parameters		
Machine Learning and Radiomics Analysis		
Comparison with known factors	73	Describe where performance of radiomics models is compared with known (clinical) factors. **
Multicollinearity	74	Describe where the multicollinearity between radiomics features in the signature is assessed. **
Model availability	75	Describe where radiomics models with the necessary pre-processing information may be found. **
Data availability	76	Describe where imaging data and relevant meta-data used in the study may be found. **

The items we followed are marked with this symbol *, and those we did not follow are marked with this symbol **. CT, computed tomography; MRI, magnetic resonance imaging; RF, radiofrequency; DWI, diffusion-weighted imaging; ADC, apparent diffusion coefficient; ROI, region of interest.

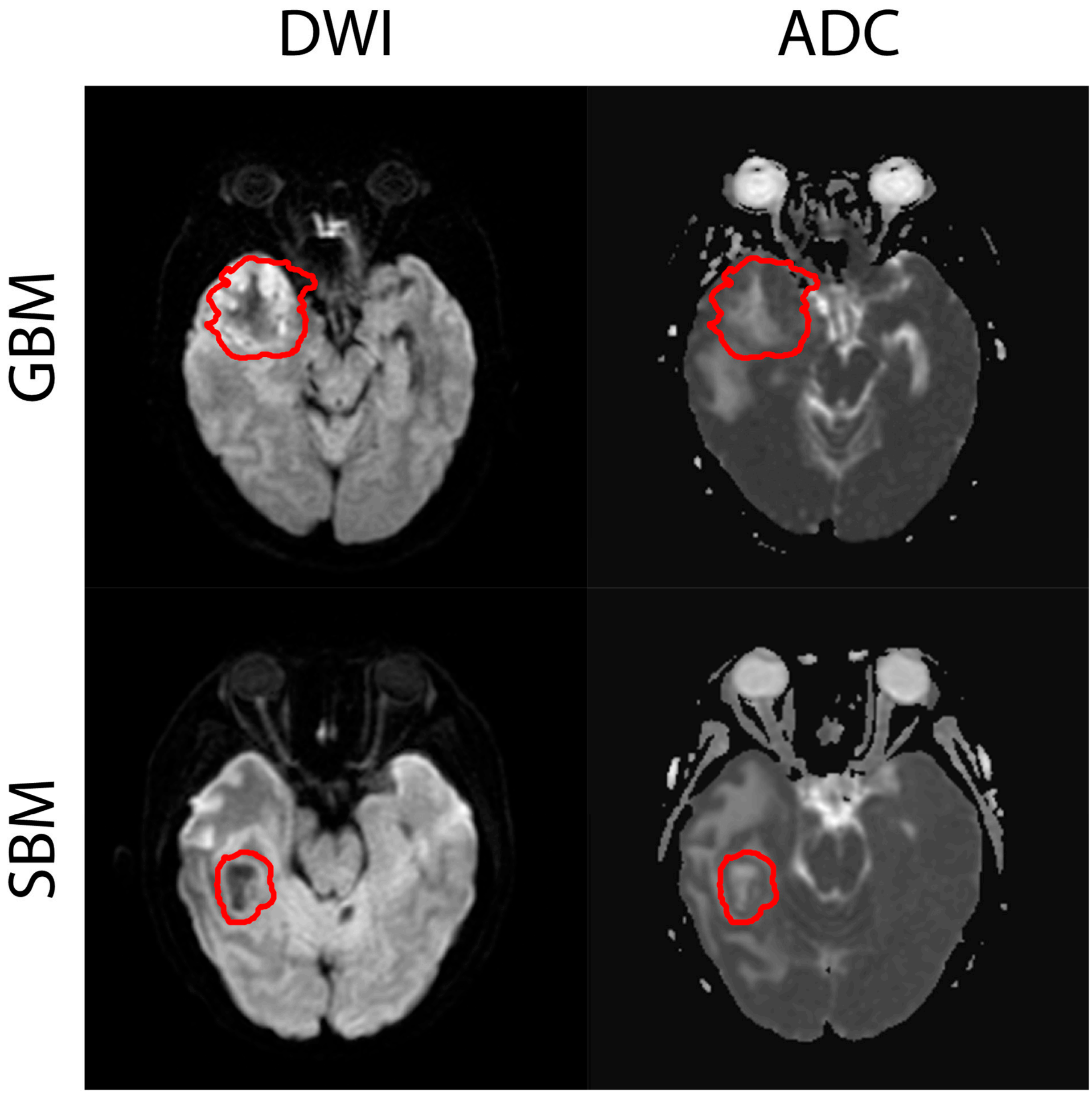

Figure S1. The representative diffusion-weighted imaging (DWI) and apparent diffusion coefficient (ADC) images from the glioblastoma multiforme (GBM) and solitary brain metastasis (SBM) patients. The enclosed regions with the red boundary were utilized for the analysis.

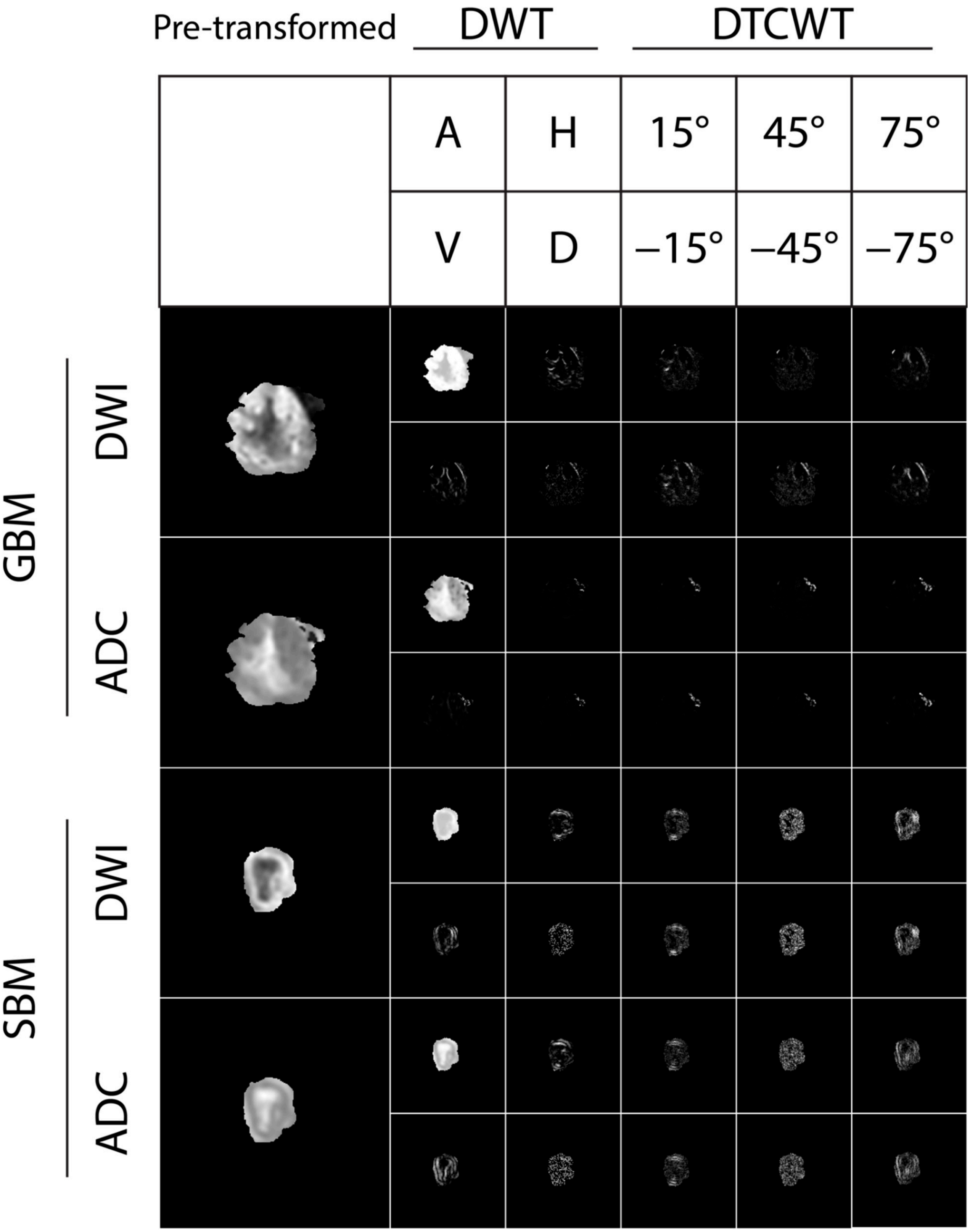

Figure S2. The enlarged region of interests (ROIs) from Figure S1 and the corresponding wavelet-transformed images. The top panels show the arrangement of the wavelet-transformed images. DWT, discrete wavelet transform; DTCWT, dual-tree complex wavelet transform; A, approximation; H, horizontal; V, vertical; D, diagonal; GBM, glioblastoma multiforme; SBM, solitary brain metastasis; DWI-MRI, diffusion-weighted imaging magnetic resonance imaging; ADC, apparent diffusion coefficient.

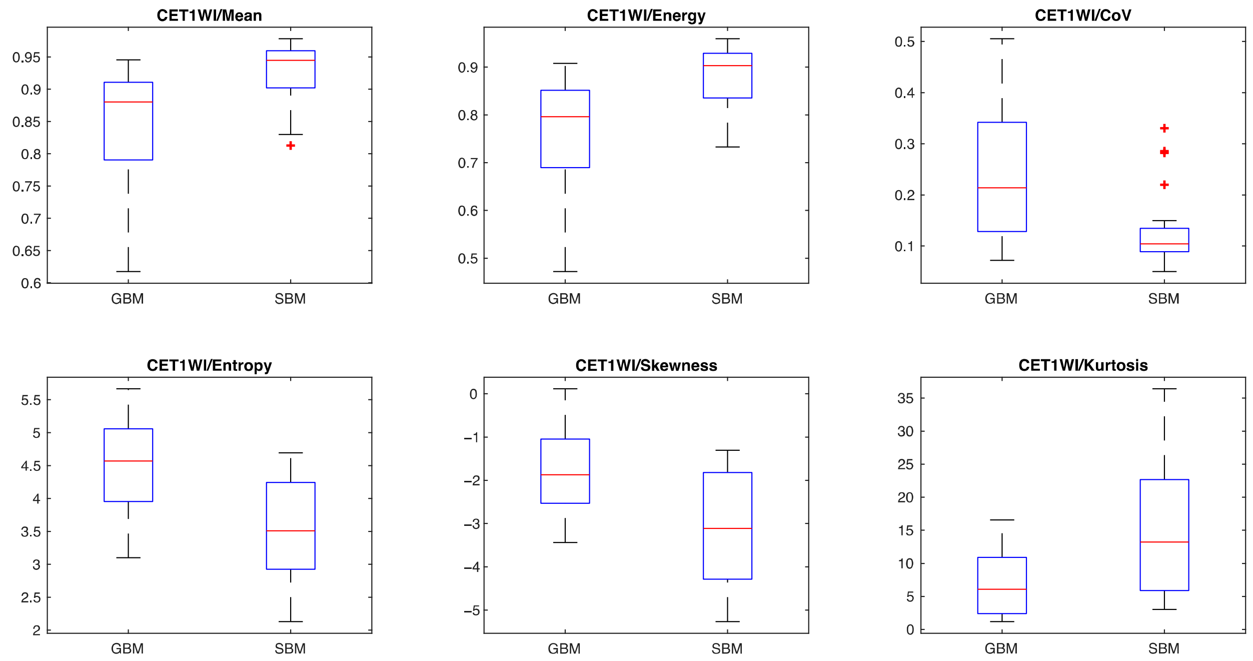

Figure S3. The box plots of the selected features from the pre-transformed images are based on the t-test ($p < 0.001$). The + sign represents the outlier. GBM, glioblastoma multiforme; SBM, solitary brain metastasis.

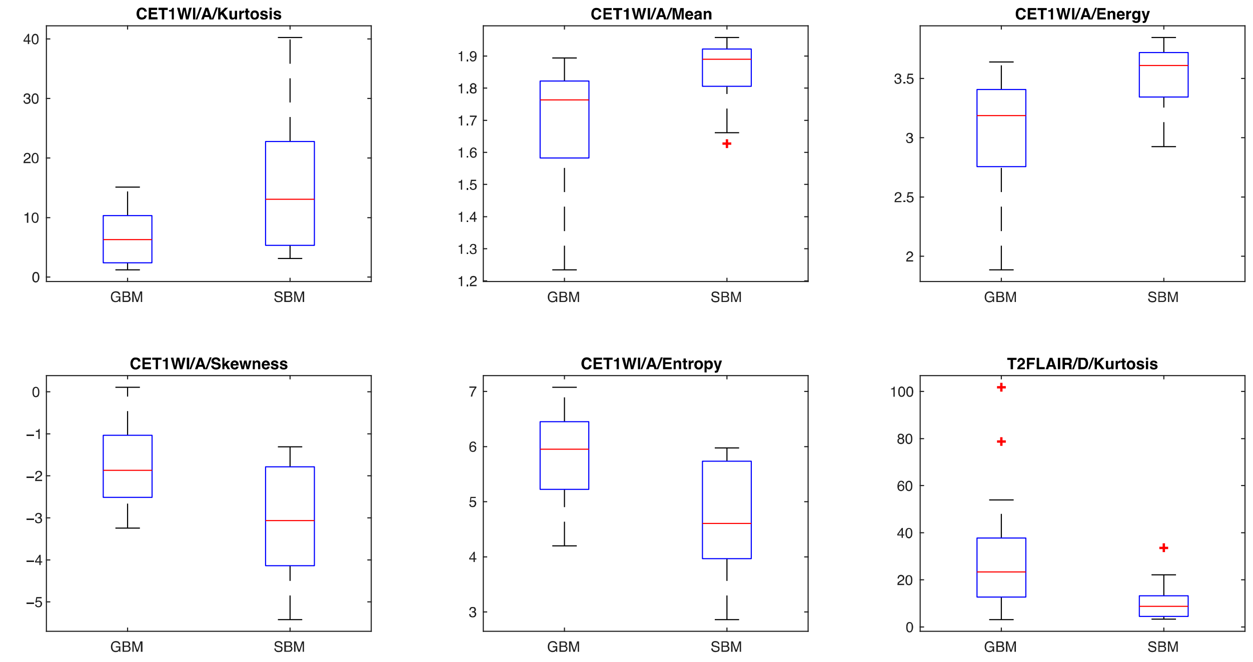

Figure S4. The box plots of the selected features from the DWT images are based on the t-test ($p < 0.001$). The + sign represents the outlier. GBM, glioblastoma multiforme; SBM, solitary brain metastasis.

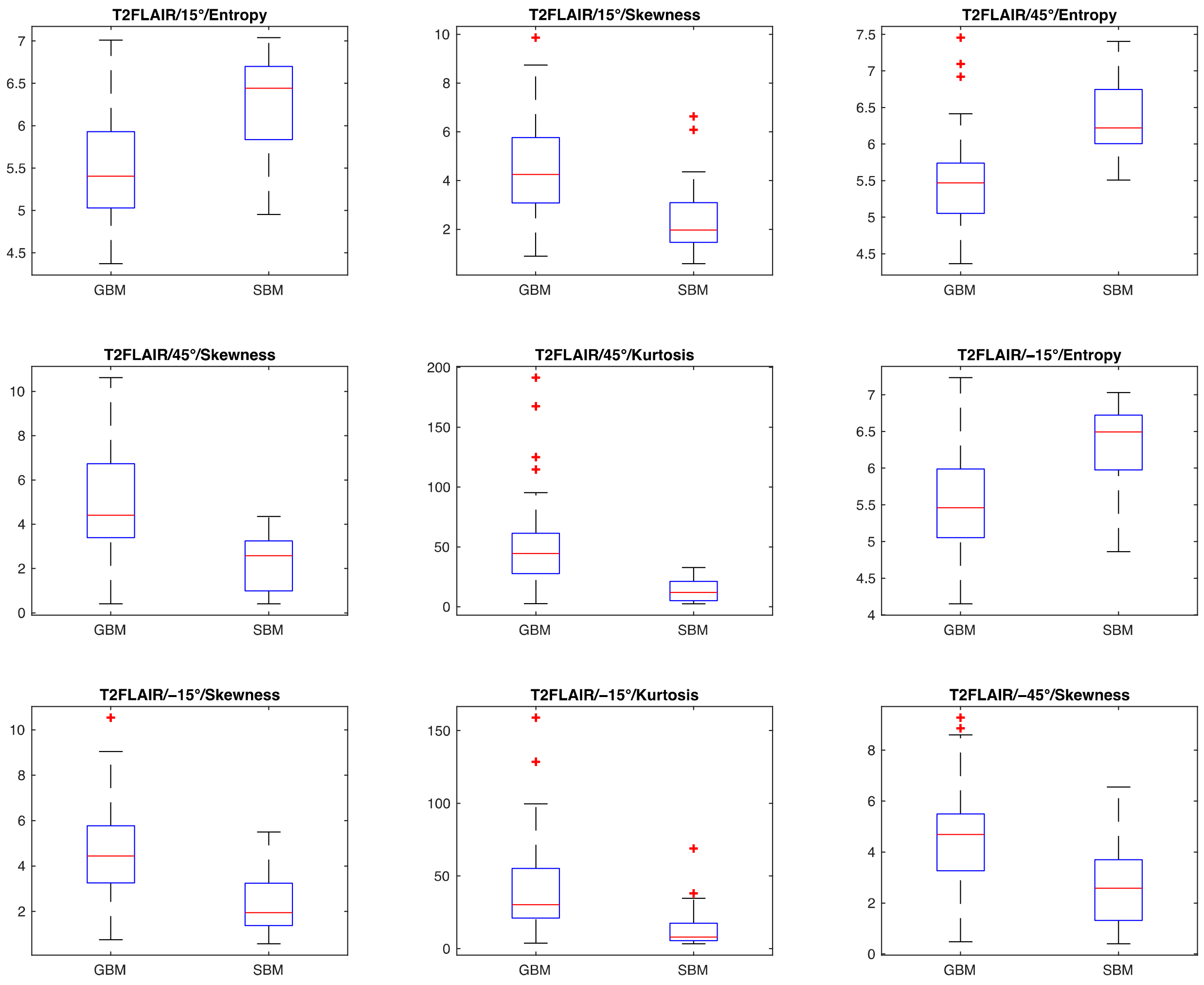

Figure S5. The box plots of the selected features from the DTCWT images are based on the t-test ($p < 0.001$). The + sign represents the outlier. GBM, glioblastoma multiforme; SBM, solitary brain metastasis.
